# Erythroid anion exchanger-1 (SLC4A1) modulates carbonic anhydrase inhibitor acetazolamide on respiration

**DOI:** 10.3389/fphys.2026.1908536

**Published:** 2026-09-10

**Authors:** Cheng-Ta Lai, Jing-Heng Lin, Tian Hu, Hui-Lin Lee, Shih-Wei Wang, Kate Hsu

**Affiliations:** 1 Department of Medicine, Mackay Medical University, New Taipei City, Taiwan; 2 Division of Colon and Rectal Surgery, Department of Surgery, MacKay Memorial Hospital, Taipei, Taiwan; 3 The Laboratory of Immunogenetics, Department of Medical Research, MacKay Memorial Hospital, New Taipei City, Taiwan; 4 Graduate Institute of Biomedical Sciences, College of Medicine, MacKay Medical University, New Taipei City, Taiwan; 5 School of Pharmacy, College of Pharmacy, Kaohsiung Medical University, Kaohsiung, Taiwan; 6 MacKay Junior College of Medicine, Nursing, and Management, New Taipei City, Taiwan

**Keywords:** acetazolamide, anion exchanger-1 (AE1; band 3), carbonic anhydrase (CA), GP.Mur blood type (Mi.III), hypercapnia, whole body plethysmography (WBP)

## Abstract

**Introduction:**

Clinically widely used acetazolamide is a potent carbonic anhydrase (CA) inhibitor. CA accelerates CO_2(g)_⇌HCO_3_^-(aq)^ bidirectional conversion. CAII, the major functional isoform, primarily resides inside red blood cells (RBCs). Erythrocyte-specific anion transporter or anion exchanger-1 (AE1) determines HCO_3_^-^ permeability across the cell membrane and is considered the rate-limiting factor for intraerythrocytic CO_2(g)_/HCO_3(aq)_ conversion facilitated by CAII. This study aimed to use CA-inhibiting acetazolamide to find out whether AE1 could modulate intraerythrocytic CA catalysis.

**Methods:**

To explore whether erythroid AE1 could modulate the respiratory effects of acetazolamide, we utilized the GPMur mouse model characteristic of higher erythroid AE1 expression for respiratory measurements using whole-body plethysmography (WBP). The effects of acetazolamide and hypercapnia on murine respiration were compared.

**Results:**

We found that acetazolamide influenced respiration in the same direction as hypercapnia. Increased erythroid AE1 expression (GPMur) counteracted the impacts of hypercapnia/acetazolamide. Importantly, GPMur/higher AE1 increased respiratory sensitivity and limited responses to acetazolamide.

**Conclusion:**

Both acetazolamide and hypercapnia drove acidosis. GPMur/increased AE1 reduced the impacts of acidosis with faster respiratory responses. This supports that AE1 and CAII function in concert to facilitate intraerythrocytic CO_2_/HCO_3_^-^ conversion for CO_2_ expiration. Since murine RBC-AE1 could help sensitize respiratory responses to acetazolamide-induced systemic CA inhibition, it warrants future clinical investigation to identify the appropriate dosing of acetazolamide for people with the GP.Mur blood type.

## Introduction

Acetazolamide has been used to treat a wide range of illness for over 70 years despite the fact that many of its mechanisms of action are still not completely understood. Acetazolamide inhibits carbonic anhydrases (CA) and reduces the rate of CO_2(g)_/HCO_3(aq)_ conversion. CA isoforms are ubiquitously present in different tissues (i.e., erythrocytes, eyes, lungs, kidneys, and other gastrointestinal organs as well as choroid plexus, and various brain cells (Lindskog, 1997; Purkerson and Schwartz, 2007)), and thus the inhibiting effects of acetazolamide could be global. Acetazolamide stimulates respiration and remains the first-line prophylactic drug for acute mountain sickness (AMS). Acetazolamide may be used as an add-on medication to treat metabolic alkalosis, particularly diuretic-induced or chloride-resistant cases; it enhances bicarbonate excretion when conventional electrolyte and volume repletion are insufficient or impractical (Mazur et al., 1999; Lopez et al., 2016). Acetazolamide also functions as a diuretic, which has been used to lower intraocular pressure (IOP) in glaucoma, to relieve special types of migraine associated with idiopathic intracranial hypertension or CADASIL (cerebral autosomal dominant arteriopathy with subcortical infarcts and leukoencephalopathy) (Marik et al., 1991; Moviat et al., 2006; Leaf and Goldfarb, 2007; Swenson and Teppema, 2007; van Patot et al., 2008; Donnini et al., 2012; Bulli et al., 2021), and to help decongestion in acute heart failure with volume overload (Kassamali and Sica, 2011; Mullens et al., 2022). Currently, acetazolamide is included in the World Health Organization’s list of essential medicines.

Both types I and II of carbonic anhydrases (CAI and CAII) are abundantly present inside human red blood cells. CAII carries out most catalytic activities for CO_2_/HCO_3_^-^ conversion inside human and murine erythrocytes (Reithmeier, 2001). CAII is the sole catalytic isoform of carbonic anhydrase in murine RBCs (Lien and Lai, 1998; Swenson, 2000); this was demonstrated by CAII-deficient mice that present metabolic and respiratory acidosis (insufficient CO_2_ excretion) (Lien and Lai, 1998).

**Figure 1:**
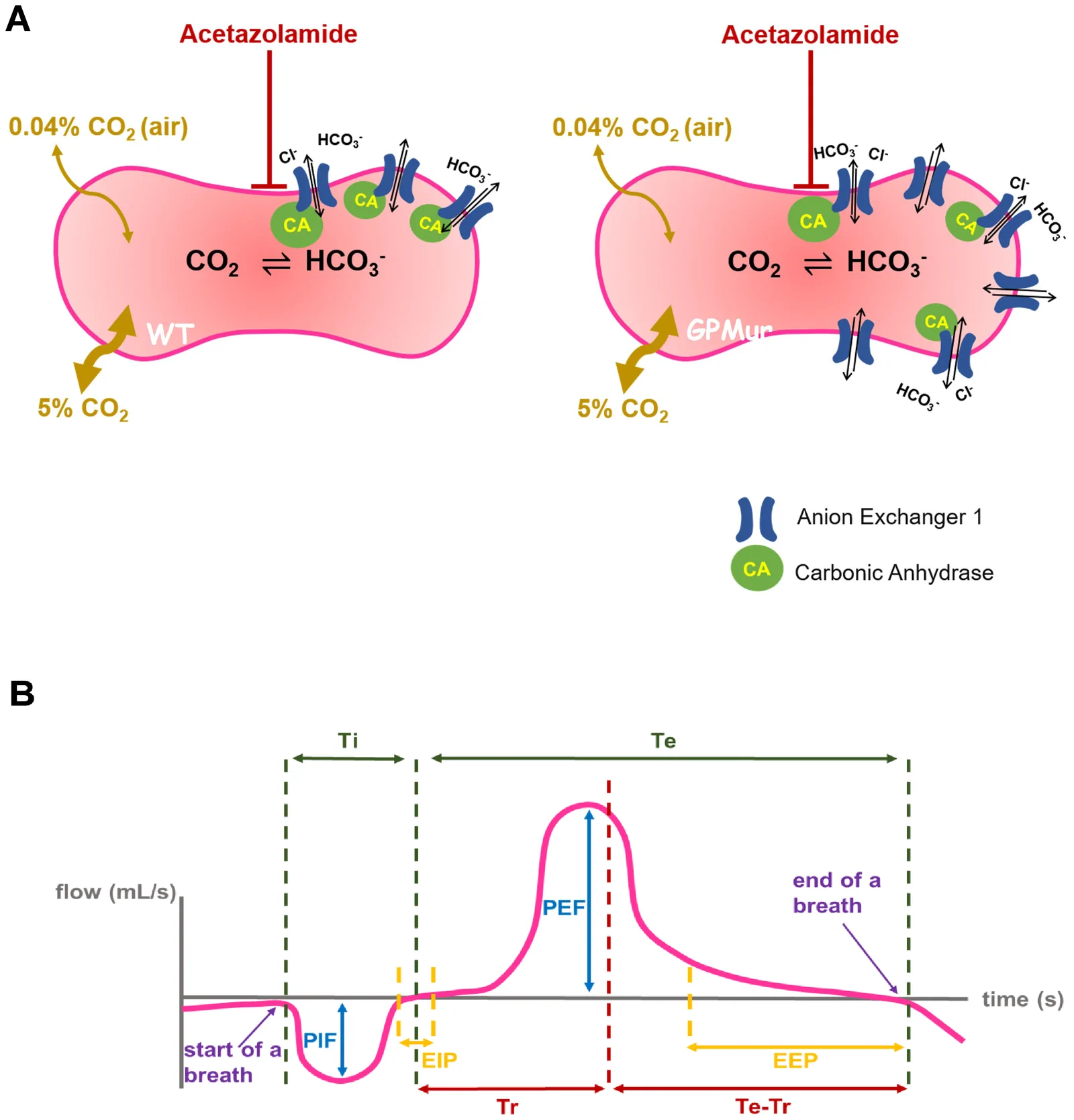
Illustrations for the rationale of the study design (top) and for special parameters from WBP (bottom). **(A)** This study employed hypercapnia (5% CO_2_) and acetazolamide challenges that directly affected the kinetics of CO_2_/HCO_3_^-^ conversion in WT versus GPMur murine RBCs and manifested on WBP measurements. **(B)** Special WBP parameters are explained in the time-flow plot for a breathing cycle (not drawn to scale). The units for the x- and y-axis are for small animals like mouse here. The definitions of WBP parameters are listed in **Supplementary Table S1**.

There are roughly ~480,000 CAII and ~1 million AE1 protein molecules in each human RBC (Moini et al., 2002). It has been suggested that CAII could directly bind to AE1 in the submembranous region, but there is also evidence against CAII–AE physical interaction (Vince and Reithmeier, 1998; Al-Samir et al., 2013). AE1 allows HCO_3_^-^ efflux or influx following the direction of the anion concentration gradients across the RBC membrane. A major source of blood HCO_3_^-^ is from CAII-catalyzed hydration of CO_2_ that takes place inside red blood cells. Because the main components that facilitate CO_2(g)_/HCO_3_^-(aq)^ conversion and HCO_3_^-^/CO_2_ transport (i.e., CAII, AE1, deoxygenated hemoglobin (deoxy Hb), and AQP1) are all in proximity of one another in the RBC membrane region, the AE1-centered multi-protein complex has been proposed to form a “CO_2_-transport metabolon (or hub)” (Vince and Reithmeier, 1998; Vince and Reithmeier, 2000; Reithmeier, 2001; Reithmeier et al., 2016; Hsu et al., 2017; Hsu, 2018). In spite of the controversy about CA–AE1 physical interaction inside RBCs (Vince and Reithmeier, 1998; Al-Samir et al., 2013), AE1 (integral membrane protein) and CAII (cytosol protein) share the same substrate HCO_3_^-^ (which is constantly converted from/to CO_2_ driven by local concentration gradients inside red blood cells); AE1 and CAII are thus functionally linked or coupled (**Figure 1A**). In their functional coupling, CAII catalytic turnover (~10^6^/s) far exceeds the capacity of AE1-mediated transport (~50,000–60,000 anions/s) (Pocker and Janjic, 1987; Pushkin et al., 2024), making AE1 the rate-limiting step. Whether AE1 could modulate CA activities or the effect of acetazolamide remains unclear.

GP.Mur is a clinically important Southeast Asian (SEA) erythrocyte type (with prevalence ranging from 1% to 2% in Malaysia and Indonesia, 9%–18% in Thailand, 6%–9.2% in Vietnam, 7%–10% in the Philippines, 18% in Eastern Taiwan, and 5%–10% in Southern China) (Prathiba et al., 2002; Palacajornsuk et al., 2007; Hsu et al., 2013a; Hsu et al., 2013b; Vongsakulyanon et al., 2015; Wei L. et al., 2016; Wei SS. et al., 2016; Ji et al., 2017; Wei et al., 2018; Hassan et al., 2019; Yang et al., 2021; Nguyen et al., 2025; Srichankhot et al., 2026) but rare in non-SEA regions (even northern Asia such as Japan, Korea, and northern China). The human *GYP.Mur* gene is a naturally occurring recombinant of erythroid *glycophorin B* (*GYPB*) and *glycophorin A* (*GYPA*) genes. Similar to glycophorin A that promotes AE1 expression, GPMur (glycophorin B-A-B hybrid), as the protein entity of human GP.Mur blood type, also enhances erythroid AE1 levels. Notably, the GPMur blood type is characteristic of 10% or higher erythroid AE1 expression (Hsu et al., 2009; Hsu et al., 2011). The higher AE1 expression accelerates transmembrane transport of HCO_3_^-^ and conceivably drives the conversion between HCO_3_^-^ and CO_2_ inside the erythrocytes (Hsu, 2018). This was supported by the fact that people with the GP.Mur blood type excrete CO_2_ more efficiently during low- and high-intensity exercise (Hsu et al., 2015; Hsu et al., 2023). The phenomenon that GPMur glycophorin B-A-B hybrid protein enhances AE1 expression on human RBCs was verified by a newly established GPMur knock-in (KI) mouse model: human GPMur protein expression in the transgenic mice increased murine erythroid AE1 by ~55% (Chen et al., 2025). Thus, in this study, we adopted the GPMur murine model to explore whether increased RBC AE1 could modulate acetazolamide’s respiratory effects by whole-body plethysmograph or WBP (**Figure 1B**).

## Methods

### *GYP.Mur* humanized knock-in mouse

Since drug-induced respiratory responses could only be studied in mammals and not organoid or cultured cells, this mouse study has been approved by the Institutional Animal Care and Use Committee (IACUC) of Taiwan MacKay Memorial Hospital (MMH-A-S-111-35-R-R) and adhered to the ARRIVE guidelines.

The *GYP.Mur* humanized KI mouse model (C57BL/6JNarl-*Gypa^eml(GYPMur)MMH^*; GPMur KI) was generated by CRISPR/Cas9-mediated DNA cleavage and homologous recombination with a replacing ssDNA on the *Gypa allele* of C57BL/6JNarl (a sub-strain bred from C57BL/6J; National Center for Biomodels [NCB], Taipei, Taiwan) as described (Chen et al., 2025). For the knock-in strategy, human *GYP.Mur* cDNA (accession EU338225) was inserted into the 3′ UTR of murine *GYPA* gene; with this strategy, the expression of human *GYP.Mur* gene was driven by the promoter of murine erythroid-specific *GYPA* gene. Knock-in of the *GYP.Mur* gene was verified by PCR sequencing using genomic DNA extracted from the tail tips of pups. GPMur KI mice produce progenies with equal male/female ratios. *GYP.Mur* knock-in drives GPMur protein expression on the murine RBC membrane and does not harm or reduce the life expectancy of C57BL6/Narl (Chen et al., 2025). The control or wild-type (WT) mice were age-matched C57BL6/Narl mice purchased from Taiwan-NCB.

### Flow cytometric measurement of CAII in mouse RBCs

The RBCs from adult male mice of both strains (C57BL/6JNarl-*Gypa^eml(GYPMur)MMH^* [GPMur KI]; C57BL/6JNarl [WT]) were washed with PBS, fixed with 1% paraformaldehyde in PBS at 4 °C for 10 min, and washed with PBS again, followed by permeabilization using 0.05% saponin in PBS/5% fetal bovine serum (FBS; Thermo Fisher Scientific, Waltham, MA, USA; catalog no. 16000044). The fixed and permeabilized RBCs were washed using PBS containing 1% bovine serum albumin (BSA) and incubated with anti-CAII antibody (1:200 dilution; GeneTex, Hsin-Chu, Taiwan) at 4 °C for 1 h, followed by 30 min of secondary antibody labeling with Alexa-Fluor-488-conjugated anti-rabbit Fab (1:200 dilution; Jackson ImmunoResearch, West Grove, PA, USA) at 4 °C in the dark. Intraerythrocytic CAII protein levels were compared based on the intensities of Alexa Fluor 488 fluorescence using FACSCalibur (BD Biosciences). The geometric mean of the WT data was set as 100%, and all data were calculated in percentile (%) relative to the geometric mean of the WT data.

### Whole-body plethysmography

From whole-body plethysmography (WBP) pressure measurements, the following ventilatory parameters were calculated: respiratory frequency (R_f_, breaths/min), tidal volume (V_T_, mL), and minute ventilation ( $\dot{V}$_E_ = R_f_ × V_T_, mL/min). In a respiratory cycle, the inspiratory time (Ti, s) and expiratory time (Te, s) can be expressed as duty cycles Ti/Ttot and Te/Ttot, where the total time of one breath or Ttot = Ti + Te. The inspiratory drive and expiratory drive are defined as V_T_/Ti and V_T_/Te (mL/s). Neural control of respiration could also be evaluated by the end-inspiratory and end-expiratory pauses (EIP and EEP, ms). The parameters relevant to expiratory kinetics are relaxation time (Tr, s) or the time for box-pressure decay to 36% of the total expiratory pressure and estimated peak expiratory and inspiratory flows (PEFb and PIFb, mL/s).

Two consecutive generations of the GPMur KI and age-matched wild-type (WT) adult male B6 mice (six mice per group × two generations) were tested for respiratory functions using Buxco^®^ WBP FinePointe (Data Sciences International) under unrestrained and conscious conditions at the core facility of the Taiwan Mouse Clinic. To follow the regulation for WBP experimentation using the DSI instrument, each WBP experiment was limited to maximally one test per week and ≤1.5 h per test to minimize the time that a study mouse was put alone in a WBP chamber.

**Figure 2:**
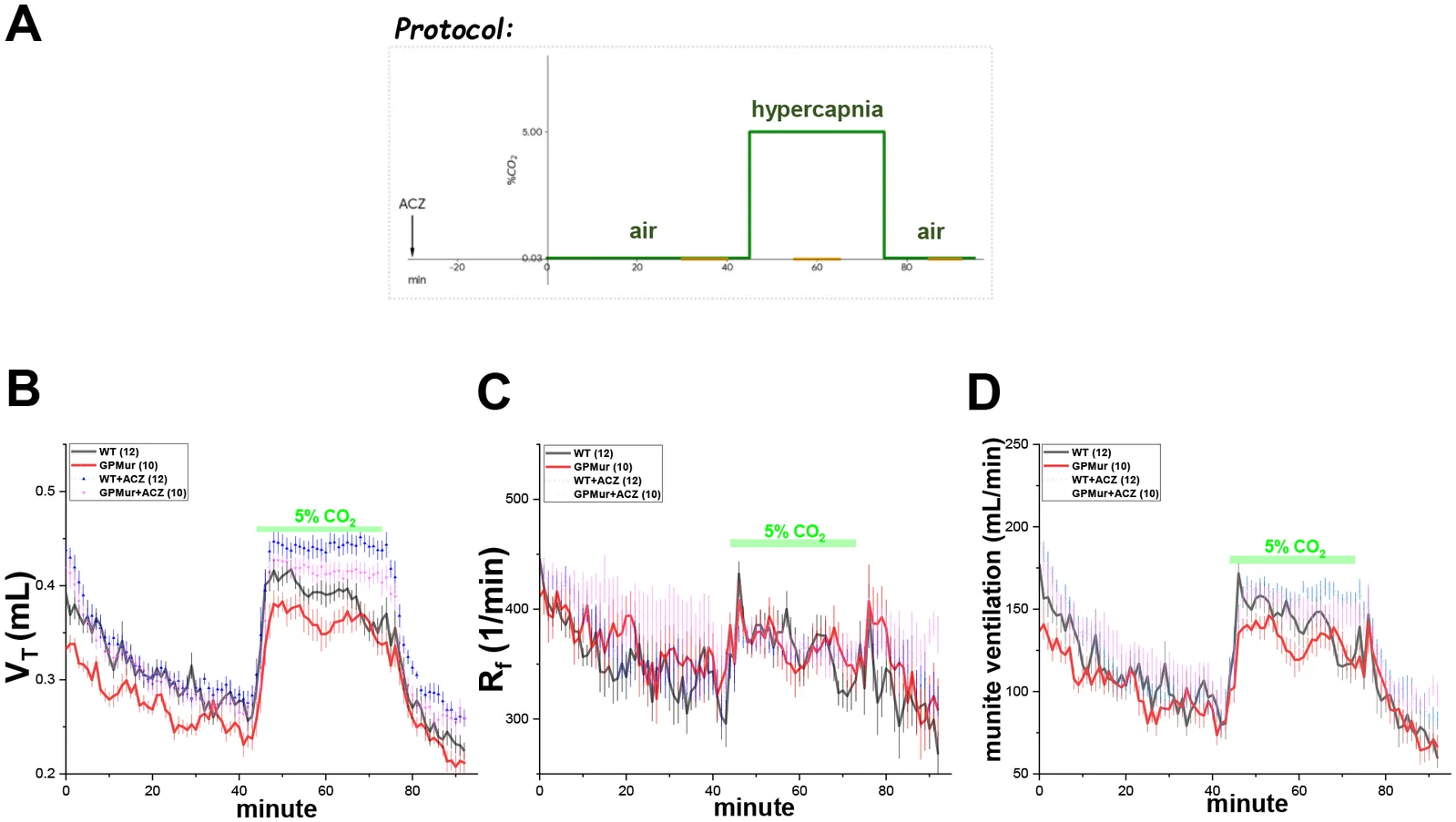
Hypercapnia increased ventilation, with sustaining effects on V_T_ and $\dot{V}$_E_. Wild-type B6 mice responded to acetazolamide only during hypercapnia, whereas GPMur KI mice responded to acetazolamide in air as well as in hypercapnia. **(A)** WBP protocol (green line). The WBP data from the two consecutive generations of homozygous GPMur male mice were consistent and were thus compiled and shown as means ± SE in **(B)** to **Figure 3** and in all **Supplementary Figures**. In each experiment, the mice were individually put in a WBP chamber with air injection (containing ~0.04% CO_2_) for 45 min (the first 30 min allowed for familiarization). From the 45th to the 75th minute, 5% of N_2_ in the injected gas was replaced by CO_2_ (hypercapnic challenge). From the 75th to the 95th minute, the injected gas was changed back to normal air. One week later, each of the mice was first administered with acetazolamide 30 min prior to the same WBP-hypercapnia test. WT and GPMur mice without acetazolamide (ACZ) pretreatments are shown in black and red; those with ACZ pretreatments are in blue and pink. The numbers of mice tested per group are indicated inside the parentheses in the legend box. **(B)** Tidal volume (V_T_). **(C)** Respiratory frequency (R_f_). **(D)** Minute ventilation ( $\dot{V}$_E_).

Murine respiratory functions were challenged by hypercapnia and/or acetazolamide. The hypercapnic challenge took place after 45-min stabilization in air flow. For the hypercapnic condition, we used premixed gas containing 5% CO_2_, 76% N_2_, and 21% O_2_. Gas-in of 5% CO_2_ has been shown to be tolerable for B6J mouse studies (Getsy et al., 2021). Each mouse was tested with the same WBP-hypercapnia protocol twice (one with the pre-treatment of acetazolamide and the other without); the two tests were scheduled 1 week apart (see **Figure 2A**—WBP protocol). To prevent habituation with hypercapnia conditioning or acetazolamide, the animals were not pre-trained in this experimental setting. Each WBP test began instead with 45 min of stabilization in air flow, which allowed individual animals to calm down in their own WBP chambers. As shown in **Figure 2A**—WBP protocol, after the WBP chamber was injected with air (~0.04% CO_2_) for 45 min (stabilization phase), pre-mixed high-CO_2_ gas was injected for 30 min (hypercapnic phase) and changed back to air (recovery phase). In the following week, the same mice were individually weighed and then given acetazolamide (50 mg/kg) by oral gavage before WBP testing. Prior to WBP, their body weights were within the normal range for adult B6 mice (GPMur KI: 29.4 ± 2.0 g; WT: 31.5 ± 2.5 g).

Compared to the typical dosage of acetazolamide for treating human patients (8–50 mg/kg/day in divided doses) (Acetazolamide; Giamello et al., 2024), a slightly higher dosage of acetazolamide (40–50 mg/kg) is generally used in rodent respiratory studies to account for human–mouse differences in metabolism and pharmacokinetics (Yamauchi et al., 2007). In our pre-test to determine an appropriate dose for WBP, we examined murine blood gas 1 h after oral gavage with different doses of acetazolamide (0–100 mg/kg BW). We found that the blood pCO_2_ between WT and the GPMur mice differed most under the dose of 50 mg/kg acetazolamide, and thus this dose was used for WBP. Others have also studied posthypoxic unstable breathing in C57BL/6 mice using 40 mg/kg acetazolamide (Yamauchi et al., 2007).

### Statistical analyses

IBM SPSS and OriginPro programs were used for all statistical computations and graphic preparations. Since WBP could be affected by multiple factors (acetazolamide, hypercapnia interval, GPMur phenotype, and body weight), linear mixed model (LMM) was used as the primary confirmation. Descriptive statistics and paired and unpaired *t-*tests were used for exploratory comparisons to characterize the data. The respiratory data collected at 30–40 min (air), 55–65 min (hypercapnia), and 85–92 min (air/recovery), respectively, were averaged and analyzed using *t*-tests and LMM. Unpaired *t-*tests were used to compare GPMur and WT groups. Paired *t-*tests were used to compare the same mice with and without acetazolamide treatments. In LMM, the hypercapnia-interval factor comprised baseline air and hypercapnia stages; the recovery stage was excluded due to large variations among animals during return to the baseline. We used the main effects model of the LMM to evaluate the overall significance of each factor; this model considers the overall effect of each factor to be independent from the other factors. To account for repeated measures of the same mouse, the ID of each mouse was included as a random effect. LMM in SPSS employs the Satterthwaite method to estimate the degrees of freedom for limited sample sizes. Statistical significance was set at *p <*0.05.

## Results

### One-fifth less CAII in murine RBC expressing GPMur

Since CAII is the main catalytically active isoform of carbonic anhydrase in human and murine RBCs (Brion et al., 1997; Lien and Lai, 1998), we first examined whether the expression of human GPMur protein could affect murine erythroid CAII levels in knock-in mice. By flow cytometric quantitation of fixed and permeabilized murine RBCs, we found ~22.4% less CAII in RBCs from GPMur KI than from WT B6 mice (77.6 ± 17.8% [GPMur, *n* = 11] *versus* 100 ± 13.6% [WT, *n* = 8]; **p = 0.009).

### GPMur/AE1-sensitized ventilatory responses to acetazolamide/hypercapnia

**Figure 3:**
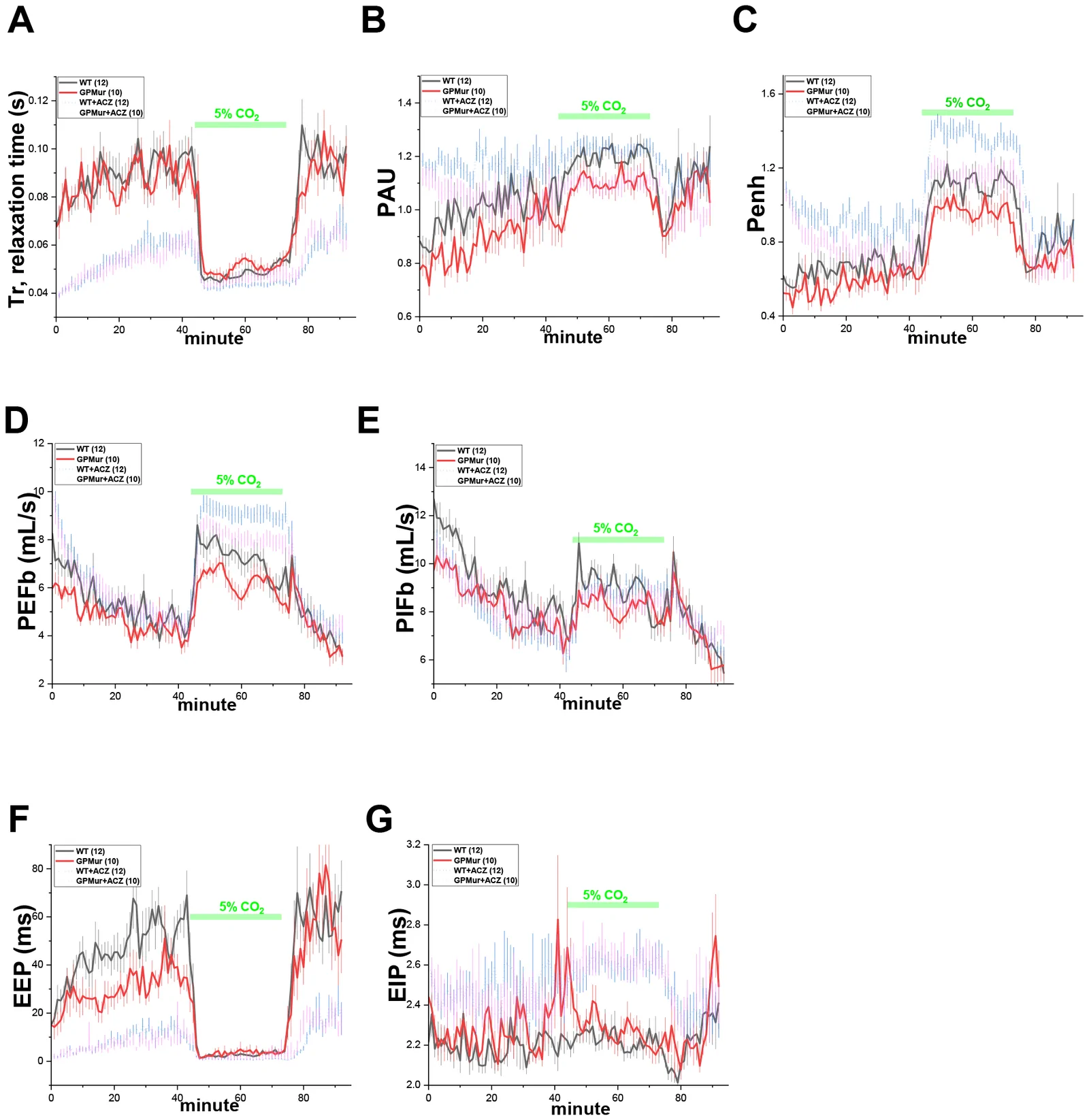
The effects of acetazolamide, hypercapnia, and GPMur phenotype on respiratory kinetics. **(A, B)** Relaxation time (T_r_) and the remaining T_e_ after subtraction of T_r_ (T_e_ – T_r_) are used to calculate pause (PAU), which is the ratio of time spent in the two expiratory phases. **(C)** Penh, or enhanced PAU, is calculated by multiplying PAU with the ratio of PEP to PIP (PAU * (PEP/PIP)). PAU and Penh were both increased by hypercapnia and acetazolamide challenges and reduced with the phenotype of GPMur/increased AE1. **(D, E)** Hypercapnia and acetazolamide significantly increased PEF throughout the entire 30-min gas-in with 5% CO_2_. In contrast, PIF was not affected by acetazolamide. **(F, G)** Hypercapnia and acetazolamide affected timing in a respiratory cycle (EEP and EIP) in the same direction.

To visualize how RBC AE1 could modulate acetazolamide’s respiratory effects on GPMur knock-in mice (**Figure 1A**) (Chen et al., 2025), we plotted the averaged traces from WBP (**Figures 2**, **3**; **Supplementary Figures**). Acetazolamide and 5% CO_2_ treatments both aimed to increase blood CO_2_ and trigger acidosis to activate central and peripheral chemoreceptors (hypercapnic ventilatory responses) (Swenson and Hughes, 1993; Vassilakopoulos, 2012). For unrestrained and unanesthetized adult C57BL/6 mice, the reported V_T_ values estimated by Quindry et al. are approximately 9 to 10 mL/kg and R_f_ 350–468/min (Quindry et al., 2016). The ventilatory data of our mice were generally near the upper ends of the reported ranges, presumably because our mice were ~20% heavier and 2 to 3 months older and/or WBP settings could vary among different studies (Bates et al., 2008; Lim et al., 2014; Quindry et al., 2016; Getsy et al., 2021).

**Table 1** summarizes the WBP-respiratory data and presents the initial exploratory analyses in the three gas-in conditions of WBP (baseline air/hypercapnia/recovery air). Acetazolamide and hypercapnia substantially affected almost all respiratory parameters in both WT and GPMur mouse strains. GPMur knock-in lowered V_T_, although its effects on respiration were mild (**Table 1**).

**Table 1:** WBP data of WT and GPMur mice upon challenge with acetazolamide (ACZ) and hypercapnia, respectively.

WBP parameter	Unit	Condition	WT (12)	WT+ACZ (12)	*P*-value (WT *vs*. WT+ACZ)^a^	GPMur (10)	GPMur+ACZ (10)	*P*-value (GPMur *vs*. GPMur+ACZ)^a^	*P*-value (WT *vs*. GPMur)	*P*-value (WT+ACZ *vs*. GPMur+ACZ)
R_f_	1/min	Air	333.9 ± 53.1	338.0 ± 40.3		353.3 ± 64.1	387.8 ± 62.6	*		*
		Hypercapnia	370.4 ± 43.4	361.5 ± 17.9		356.2 ± 41.3	364.4 ± 31.7			
		Recovery	304.5 ± 53.1	337.1 ± 24.8	*	313.5 ± 75.6	368.8 ± 46.2	*		
V_T_	mL	Air	0.277 ± 0.021	0.290 ± 0.023		0.257 ± 0.015	0.276 ± 0.028	0.1	**	
		Hypercapnia	0.393 ± 0.024	0.441 ± 0.034	**	0.360 ± 0.042	0.415 ± 0.030	**	**	0.07
		Recovery	0.237 ± 0.023	0.270 ± 0.025	**	0.220 ± 0.025	0.260 ± 0.030	**		
$\dot{V}$_E_	mL/s	Air	94.6 ± 20.9	97.7 ± 13.8		91.9 ± 17.8	107.7 ± 24.4	*		
		Hypercapnia	144.9 ± 22.3	158.8 ± 14.7	0.06	127.2 ± 17.7	150.6 ± 17.5	*	0.051	
		Recovery	73.6 ± 19.4	91.3 ± 12.4	*	70.5 ± 20.8	95.6 ± 15.5	**		
Te	s	Air	0.171 ± 0.032	0.128 ± 0.018	**	0.157 ± 0.027	0.110 ± 0.019	***		*
		Hypercapnia	0.103 ± 0.013	0.095 ± 0.005	0.05	0.106 ± 0.012	0.094 ± 0.009	**		
		Recovery	0.179 ± 0.022	0.131 ± 0.016	***	0.183 ± 0.051	0.119 ± 0.011	**		0.055
Te/Tot		Air	0.733 ± 0.027	0.650 ± 0.019	***	0.726 ± 0.020	0.644 ± 0.021	***		
		Hypercapnia	0.597 ± 0.012	0.565 ± 0.008	***	0.595 ± 0.016	0.562 ± 0.013	***		
		Recovery	0.723 ± 0.044	0.662 ± 0.029	***	0.729 ± 0.015	0.660 ± 0.031	***		
Ti	s	Air	0.061 ± 0.012	0.068 ± 0.010	*	0.059 ± 0.012	0.061 ± 0.011			
		Hypercapnia	0.069 ± 0.007	0.073 ± 0.004	0.07	0.072 ± 0.009	0.073 ± 0.007			
		Recovery	0.069 ± 0.020	0.066 ± 0.004		0.067 ± 0.018	0.061 ± 0.010			
Ti/Tot		Air	0.267 ± 0.027	0.350 ± 0.019	***	0.274 ± 0.020	0.356 ± 0.021	***		
		Hypercapnia	0.403 ± 0.012	0.435 ± 0.008	***	0.405 ± 0.016	0.438 ± 0.013	***		
		Recovery	0.277 ± 0.044	0.338 ± 0.029	***	0.271 ± 0.015	0.340 ± 0.031	***		
V_T_/Te	mL/s	Air	1.75 ± 0.46	2.38 ± 0.33	**	1.74 ± 0.30	2.63 ± 0.65	**		
		Hypercapnia	3.96 ± 0.66	4.66 ± 0.42	**	3.49 ± 0.55	4.45 ± 0.56	**		
		Recovery	1.40 ± 0.30	2.17 ± 0.39	***	1.33 ± 0.36	2.25 ± 0.44	***		
V_T_/Ti	mL/s	Air	4.78 ± 0.94	4.39 ± 0.61		4.59 ± 0.80	4.74 ± 0.96			
		Hypercapnia	5.86 ± 0.81	6.06 ± 0.59		5.13 ± 0.69	5.70 ± 0.61	0.05	*	
		Recovery	3.80 ± 1.04	4.21 ± 0.45		3.62 ± 0.98	4.34 ± 0.63	*		
PAU		Air	1.04 ± 0.18	1.19 ± 0.14	*	0.96 ± 0.14	1.02 ± 0.12			***
		Hypercapnia	1.20 ± 0.08	1.23 ± 0.07		1.10 ± 0.10	1.09 ± 0.09		*	***
		Recovery	1.15 ± 0.16	1.13 ± 0.12		1.10 ± 0.14	1.00 ± 0.10	0.08		*
Penh		Air	0.673 ± 0.169	0.876 ± 0.128	**	0.621 ± 0.110	0.714 ± 0.124			***
		Hypercapnia	1.10 ± 0.16	1.38 ± 0.12	***	0.96 ± 0.16	1.15 ± 0.14	**	0.055	***
		Recovery	0.832 ± 0.249	0.836 ± 0.128		0.727 ± 0.093	0.713 ± 0.133			*
Tr	s	Air	0.094 ± 0.018	0.062 ± 0.007	***	0.090 ± 0.011	0.057 ± 0.009	***		
		Hypercapnia	0.048 ± 0.006	0.043 ± 0.003	0.05	0.052 ± 0.006	0.046 ± 0.006	*		
		Recovery	0.093 ± 0.013	0.065 ± 0.012	***	0.095 ± 0.016	0.063 ± 0.006	**		
PEFb	mL/s	Air	4.63 ± 1.21	4.74 ± 0.72		4.35 ± 0.86	4.82 ± 1.10			
		Hypercapnia	7.30 ± 1.42	9.07 ± 1.06	***	6.06 ± 1.14	8.05 ± 0.86	**	*	*
		Recovery	3.83 ± 0.87	4.40 ± 0.68	0.11	3.49 ± 1.01	4.35 ± 0.83	*		
PIFb	mL/s	Air	8.00 ± 1.45	7.12 ± 1.02	0.06	7.48 ± 1.21	7.63 ± 1.53			
		Hypercapnia	9.08 ± 1.23	8.91 ± 0.87		8.01 ± 0.94	8.44 ± 0.98		*	
		Recovery	6.42 ± 1.72	6.78 ± 0.64		6.10 ± 1.51	6.98 ± 0.98	0.11		
EEP	ms	Air	54.3 ± 15.8	16.2 ± 12.6	***	36.9 ± 9.1	9.9 ± 8.6	***		
		Hypercapnia	2.56 ± 1.05	0.83 ± 0.44	***	3.92 ± 4.99	1.58 ± 3.41	**	**	
		Recovery	59.2 ± 22.1	19.2 ± 10.8	***	63.6 ± 47.7	17.0 ± 9.5	*		
EIP	ms	Air	2.20 ± 0.09	2.49 ± 0.22	**	2.28 ± 0.16	2.40 ± 0.25			
		Hypercapnia	2.22 ± 0.11	2.59 ± 0.16	***	2.26 ± 0.17	2.63 ± 0.20	***		
		Recovery	2.33 ± 0.19	2.36 ± 0.19		2.38 ± 0.24	2.43 ± 0.27			

Individual WBP data from the three time intervals (1 [30~40 min], 2 [55~65 min], and 3 [85~92 min]) were averaged and shown in mean±SD. WT and GPMur mice were compared by unp1ed two-sample t-tests. aData from the same mice with and without ACZ treatments were compared by p1ed t-tests. *P-values < 0.05, **P-values < 0.01 denoted **, ***P-values < 0.001. The number of mice tested per group is indicated inside the parenthesis.

We used LMM as the confirmatory analyses which accounted for all four factors (hypercapnia, acetazolamide, GPMur expression, and body weight [BW]) and repeated measures of the same mice and found that (1) hypercapnia and acetazolamide were predominant factors influencing WBP-respiratory parameters and (2) GPMur still showed significant lowering effects on V_T_ (**Figure 2**; **Table 2**). The latter is consistent with previous findings from the cardiopulmonary exercise testing (CPET) of college athletes: GP.Mur+ athletes persistently showed lower V_T_ throughout CPET (Hsu et al., 2023). Differently from GP.Mur+ athletes who showed significantly higher R_f_ and larger $\dot{V}$_E_ during CPET, here unrestrained GPMur and WT mice inside WBP chambers did not differ significantly in R_f_. This could be due to a lack of strong physical stimulation (e.g., running on a treadmill) and large R_f_ fluctuation associated with the hypersensitive nature of the B6J strain (lowering the statistical power).

**Table 2:** Assessing the effects of the four variables—body weight, GPMur phenotype, acetazolamide treatment, and CO_2_ increase to 5% (hypercapnia)—on murine respiratory parameters by linear mixed models (LMM main effects).

Effects on murine WBP parameters	Body weight		GPMur type		Acetazolamide		Increase to 5% CO_2_	
	*p*-value	Effects	*p*-value	Effects	*p*-value	Effects	*p*-value	Effects
R_f_ (/min)	n.s.		n.s.		n.s.		n.s.	
V_T_ (mL)	*p* < 0.001	↑	*p* < 0.05	↓	*p* < 0.001	↑	*p* < 0.001	↑
$\dot{V}$_E_ (mL/min)	n.s.		n.s.		*p* < 0.01	↑	*p* < 0.001	↑
Te (s)	n.s.		n.s.		*p* < 0.001	↓	*p* < 0.001	↓
Te/Ttot	n.s.		n.s.		*p* < 0.001	↓	*p* < 0.001	↓
Ti (s)	n.s.		n.s.		n.s.		*p* < 0.001	↑
Ti/Ttot	n.s.		n.s.		*p* < 0.001	↑	*p* < 0.001	↑
VT/Te	n.s.		n.s.		*p* < 0.001	↑	*p* < 0.001	↑
VT/Ti	n.s.		n.s.		n.s.		*p* < 0.001	↑
PAU	*p* < 0.01	↑	*p* < 0.001	↓	n.s.		*p* < 0.001	↑
Penh	*p* < 0.001	↑	*p* < 0.01	↓	*p* < 0.001	↑	*p* < 0.001	↑
Tr (s)	n.s.		n.s.		*p* < 0.001	↓	*p* < 0.001	↓
PEFb (mL/s)	n.s.		n.s.		*p* < 0.001	↑	*p* < 0.001	↑
PIFb (mL/s)	n.s.		n.s.		n.s.		*p* < 0.001	↑
EEP (ms)	n.s.		n.s.		*p* < 0.01	↓	*p* < 0.001	↓
EIP (ms)	n.s.		n.s.		*p* < 0.001	↑	*p* < 0.05	↑

*P >*0.05 (statistically not significant) is denoted n.s. The direction of the effects of a variable is determined from “estimates of fixed effects” in LMM and is indicated by an upward arrow for increasing effects or a downward arrow for decreasing effects.

### Expiratory kinetics affected by acidosis and GPMur/AE1 in opposite directions

Expiratory kinetics was characterized by relaxation time (T_r_) and peak expiratory/inspiratory flow (PEF/PIF) (**Figure 3**). Acetazolamide and hypercapnia both triggered acidosis and significantly increased PEF and PIF, indicating a need for high airflow to compensate respiratory adjustments and support acid–base homeostasis. T_r_ is directly derived from the measured volume curve and is defined as the time from the start of expiration until the time that two-thirds of the tidal volume has been exhaled in a respiratory cycle. In a normal passive exhalation, T_r_ is essentially the time constant of lung exhalation; prolonged T_r_ may mean an increase of airway resistance.

The definition of T_r_ and (T_e_ - T_r_) reflects kinetically distinct early and late expiratory phases in a breathing pattern (**Figure 1B**). PAU compares the time on the early, active part of exhalation (T_r_) to the later part of exhalation (T_e_ - T_r_) in a breath and is defined as the ratio of (T_e_ - T_r_) to T_r_. Higher PAU means that the mouse may be in labored breathing, as the animal is spending a longer time in the later than the early active stage of exhalation. Penh is PAU times the ratio of the peak expiratory flow to the peak inspiratory flow (PEF/PIF). PAU is a value of expiratory kinetics, whereas Penh reflects not just expiratory kinetics but also waveforms. Both hypercapnic and acetazolamide challenges increased Penh and PAU; in contrast, GPMur expression persistently lowered Penh and PAU (**Table 2**, **Figures 3A–C**). Although whether Penh could be used to assess airway broncoconstriction is controversial (Hamelmann et al., 1997), the observation that GPMur/increased AE1 significantly affected murine expiratory kinetics (**Table 2**) is reminiscent of similar findings from the human GP.Mur CPET study (Hsu et al., 2023).

Finally, we probed into neural control of breathing rhythm. Both hypercapnia and acetazolamide shortened expiratory time (T_e_) and its fraction in a respiratory cycle (T_e_/T_tot_), while T_i_ and T_i_/T_tot_ were increased (**Supplementary Figure 2**). This was driven by the need to increase tidal volume and ventilation upon the onset of acidosis. Both stimulations increased expiratory drive and inspiratory drives (VT/T_e_ and VT/T_i_, respectively) in the unrestrained mice (**Supplementary Figure 3**). Indeed hypercapnia has been reported to trigger active expiration to expand tidal volume by shortening T_e_ and T_e/Ttot_ in unanesthetized rodents (Leirao et al., 2018).

Together with shortened T_e_ and lengthened T_i_ (**Supplementary Figure 2**), both hypercapnia and acetazolamide substantially shortened end-expiratory pause (EEP: the duration from the end of expiration to the beginning of inspiration) and elongated end-inspiratory pause (EIP: the duration from the end of inspiration to the beginning of expiration) (**Figures 3F, G**). Although both triggers of acidosis (acetazolamide and 5% CO_2_) affected EEP and EIP in the same direction, their effects on the neural timing of respiration could be differentiated, as shown in **Figures 3F, G**. Hypercapnia significantly shortened EEP for the entire 30 min and transiently prolonged EIP. For comparison, acetazolamide shortened EEP and increased EIP substantially, unlike the transient effects of hypercapnia on EIP. Additionally, these data revealed rapid neuronal responses to acetazolamide by respiratory kinetic adjustments; this has implications for the drug mechanisms underlying AMS.

## Discussion

The whole-body plethysmography study examined the different impacts of erythroid AE1 with acidosis triggers (acetazolamide and hypercapnia) on respiration. We found that acetazolamide elicited earlier ventilatory responses in GPMur knock-in than WT mice. The lower tidal volume in GPMur mice (**Figure 2**; **Supplementary Figure 1**), consistent with GP.Mur+ people, may reflect more efficient CO_2_ excretion per breath due to enhanced erythroid AE1-mediated HCO_3_^-^ transport. Ventilation with smaller tidal volumes potentially reduces the work of breathing. In human athletes, this is compensated by increased respiratory rates, resulting in higher overall minute ventilation (Hsu et al., 2023). By increasing blood CO_2_ and acidosis (verified by blood gas measurements), the two stimuli (hypercapnia and acetazolamide) substantially enlarged minute ventilation (Swenson, 2000). Notably, acetazolamide stimulation in both strains of the B6 mice manifested in persistent V_T_ expansion and not in transient bursts of the respiratory rates (**Figure 2**; **Supplementary Figure 1**). This verifies others’ findings that acetazolamide predominantly expands tidal volume to increase minute ventilation (Tojima et al., 1986).

In these WBP experiments, the effects of GPMur/increased AE1 were opposite to the effects of acidosis by acetazolamide or hypercapnia (**Table 2**). Both acetazolamide and hypercapnia increased V_T_, minute ventilation ( $\dot{V}$E), inspiratory time (Ti), inspiratory duty cycle (Ti/Ttot), expiratory drive (VT/Te), estimated peak expiratory flow (PEFb), and end inspiratory pause (EIP); both also decreased expiratory time (Te), expiratory duty cycle (Te/Ttot), relaxation time (Tr), and end expiratory pause (EEP). In contrast, GPMur mice showed lowered tidal volume (V_T_) and distinct expiratory kinetics. The altered expiratory kinetics in GPMur mice likely reflects enhanced CO_2_ excretion rates at the alveolar–capillary interface. Faster CO_2_ unloading in pulmonary capillaries is a consequence of accelerated erythroid CO_2_/HCO_3_^-^ conversion facilitated by AE1 transport; these alter sensing of central and peripheral chemoreceptors that control breathing patterns.

Respiratory control involves peripheral (RBC and carotid body chemoreceptors) and central (brainstem chemoreceptors) components. The present WBP study focuses on erythroid HCO_3_^-^ transport. It has been reported that H^+^-sensing central chemoreceptors in the brainstem respiratory center contribute 70%–80% of the hypercapnic ventilatory responses (HVR), and peripheral carotid chemoreceptors contribute only 20%–30% HVR (Vassilakopoulos, 2012). However, the contribution of RBC had not been clear. Here the faster ventilatory responses of GPMur mice demonstrated the RBC-AE1 contribution to respiration (opposite direction to that of acidosis). Thus, the effect of RBC-AE1 through CO_2_/HCO_3_^-^ conversion should be viewed as an integral part of systemic respiratory control.

From the perspective of molecular mechanism, these findings support that AE1-mediated HCO_3_^-^ transport, rather than CAII catalysis, is the rate-limiting step in erythroid AE1–CA functional coupling, despite ~22% less CAII in murine GPMur than WT RBCs. This interpretation is grounded in the hypothesis of “erythrocyte CO_2_-transport metabolon,” in which AE1, GPA/GPB (GPMur), Rh/RhAG, AQP1, deoxyhemoglobin (deoxyHb), and CAII are physically or indirectly associated with one another in close proximity on the RBC membrane (Reithmeier, 2001; Bruce et al., 2003). The interaction between AE1 complexes and cytosol-abundant deoxyHb and CAII is transient. Erythroid AE1 and CAII share the substrate HCO_3_^-^, and yet erythroid CAII catalytic turnover (~10^6^/s) far exceeds AE1 transport capacity (~10^4^–10^5^/s) (Pocker and Janjic, 1987); therefore, AE1-mediated HCO_3_^-^ membrane transport is the rate-limiting step in their functional coupling (Reithmeier, 2001). In this study, increased erythroid AE1 drove faster ventilatory responses despite reduced CAII in murine GPMur RBCs (**Figure 1**); this again points to AE1 (not CAII) as the rate-limiting factor in blood CO_2_ metabolism. Nonetheless, an alternative explanation should be considered. Because acetazolamide-mediated CA inhibition is systemic and beyond RBC, any strain/species differences in CA tissue distribution and acetazolamide pharmacokinetics might also contribute to the indirect effects of acetazolamide or compensatory mechanisms. We favor AE1 being rate-limiting because it is the only explanation reconciling the faster ventilatory responses in GPMur mice and GP.Mur+ individuals (Hsu et al., 2015; Hsu et al., 2023).

Besides AE1 and CAII, Rhesus-associated glycoprotein (RhAG), AQP1, and UT-B on the erythrocyte membrane also participate in the transport of CO_2_ and H_2_O. RhAG is a gas channel that primarily transports NH_3_ over CO_2_ (Endeward et al., 2008; Musa-Aziz et al., 2009). Erythroid AQP1 is responsible for ~85% of RBC osmotic water permeability and ~60% of RBC CO_2_ permeability (the latter likely through the dual pathways of AQP1) (Endeward et al., 2008; Musa-Aziz et al., 2009; Musa-Aziz et al., 2026). The urea transporter UT-B (SLC14A1; the Kidd/Jk antigen) mainly transports urea, but it also transports H_2_O and NH_3_; the physiological significance for the transport of the latter two substrates remains unclear (Yang and Verkman, 2002; Azouzi et al., 2013; Geyer et al., 2013; Leifelt et al., 2024). Among these three erythroid transporters, the expression of UT-B (~14,000 copies per RBC) is less than 1/10 of the levels of RhAG (~100,000–200,000 copies) or AQP1 (~200,000 copies) (Anstee, 1990; Bryk and Wisniewski, 2017). RhAG is physically associated with tetrameric AE1 macrocomplexes (Bruce et al., 2003; Hsu et al., 2009). AQP1 transiently interacts with AE1 on the RBC membrane, as revealed by cryo-EM and FLIM-FRET (Hsu et al., 2017; Vallese et al., 2022). Conceivably, GP.Mur-enhanced AE1 expression may remodel the RBC membrane that, in principle, could alter the abundance or organization of other transporters/channels. Indeed we previously found fewer Rh/RhAG complexes in GP.Mur^+/+^ human RBCs (Hsu et al., 2012). Their different expressions and potential co-regulation on the RBC membrane are directions for future work.

Finally, the GPMur knock-in model recapitulates the direction of the human GP.Mur phenotype, namely: (1) human GPMur protein expression increases murine erythroid AE1 in mice and men (Hsu et al., 2009; Hsu et al., 2011; Chen et al., 2025), (2) here the mice reproduce ventilatory patterns (lower tidal volume and altered expiratory kinetics) documented in GP.Mur+ people during exercise (Hsu et al., 2015; Hsu et al., 2023), and (3) both GPMur+ mice and men have a higher blood pressure that is related to nitric oxide metabolism (Hsu et al., 2021; Chen et al., 2025). The first two warrant further translational–clinical investigation (e.g., blood gas assessment of acetazolamide dose responses) to verify whether GP.Mur+ individuals could achieve equivalent respiratory effects at lower doses.

### Limitation

First, this is a murine WBP study using GPMur knock-in mice characteristic of ~55% higher erythroid AE1 expression. The magnitudes of the observed respiratory effects may not be translated directly to humans (10%–20% higher erythroid AE1in GP.Mur+ people). Second, murine RBCs lack CAI isoform that is present in human RBCs. Although the role of CAI in RBC is unclear, whether erythroid AE1–CA functional coupling could be affected by different CA isoforms is also unknown. Third, CA is ubiquitously expressed, while AE1 expression is restricted to the erythroid system.

It is thus unknown whether AE1 through circulating red blood cells could remotely influence carbonic anhydrases in other systems, such as the brainstem respiratory center, peripheral chemoreceptors, and/or kidneys. As acetazolamide systemically inhibits CA, the present WBP study design could not separate the drug effects on erythrocytes *versus* on other organs. Lastly, WBP inferred chemoreceptor and pulmonary responses from ventilatory-pressure measurements rather than direct measurements of chemoreceptor activity or alveolar–capillary CO_2_ exchange. Considering that respiratory mechanics and chemoreflex set points may vary between mice and men, how erythroid AE1 influences respiratory neuronal/chemoreceptor responses remains to be explored.

## Conclusion

From this murine WBP study of conscious, unrestrained breathing, we found that increased RBC-AE1 expression (the GPMur phenotype) enhanced acetazolamide sensitivity in respiration. This implies that GP.Mur+ individuals may achieve therapeutic respiratory effects at lower acetazolamide doses and thus warrants future clinical investigation.

## Data Availability

The original contributions presented in the study are included in the article/**Supplementary Material**. Further inquiries can be directed to the corresponding author.
