# Supplementary material for: Erythroid anion exchanger-1 (SLC4A1) modulates carbonic anhydrase inhibitor acetazolamide on respiration
Source: Front Physiol. 2026 Sep 10;17:1908536. doi: 10.3389/fphys.2026.1908536 (PMC13600823; doi:10.3389/fphys.2026.1908536)

Table 1. **Major WBP-derived ventilatory parameters.**

parameters	***definition***
**R_f (/min)**	respiratory frequency
**V_T (mL)**	tidal volume
**$\dot{V}_E$ (mL/min)**	minute ventilation
**Te (s)**	expiratory time
**Te/Ttot**	time fraction of expiration in a respiratory cycle, or expiratory duty cycle
**Ti (s)**	inspiratory time
**Ti/Ttot**	time fraction of inspiration in a respiratory cycle, or inspiratory duty cycle
**VT/Te**	expiratory drive
**VT/Ti**	inspiratory drive
**Tr (s)**	relaxation time, or the time for the box pressure decay to 36% of the total pressure signals during expiration
**PEFb (mL/s)**	estimated peak expiratory flow
**PIFb (mL/s)**	estimated peak inspiratory flow
**EEP (ms)**	end expiratory pause
**EIP (ms)**	end inspiratory pause

<u>Supplemental Figure 1.</u>. Hypercapnia increased ventilation, with sustaining effects on V_T and $\dot{V}_E$. Wildtype B6 mice responded to acetazolamide only during hypercapnia, whereas GPMur KI mice responded to acetazolamide in air as well as in hypercapnia. The (A) WBP protocol (green line). (B) Tidal volume (V_T); (C) respiratory frequency (R_f); (D) minute ventilation ($\dot{V}_E$).

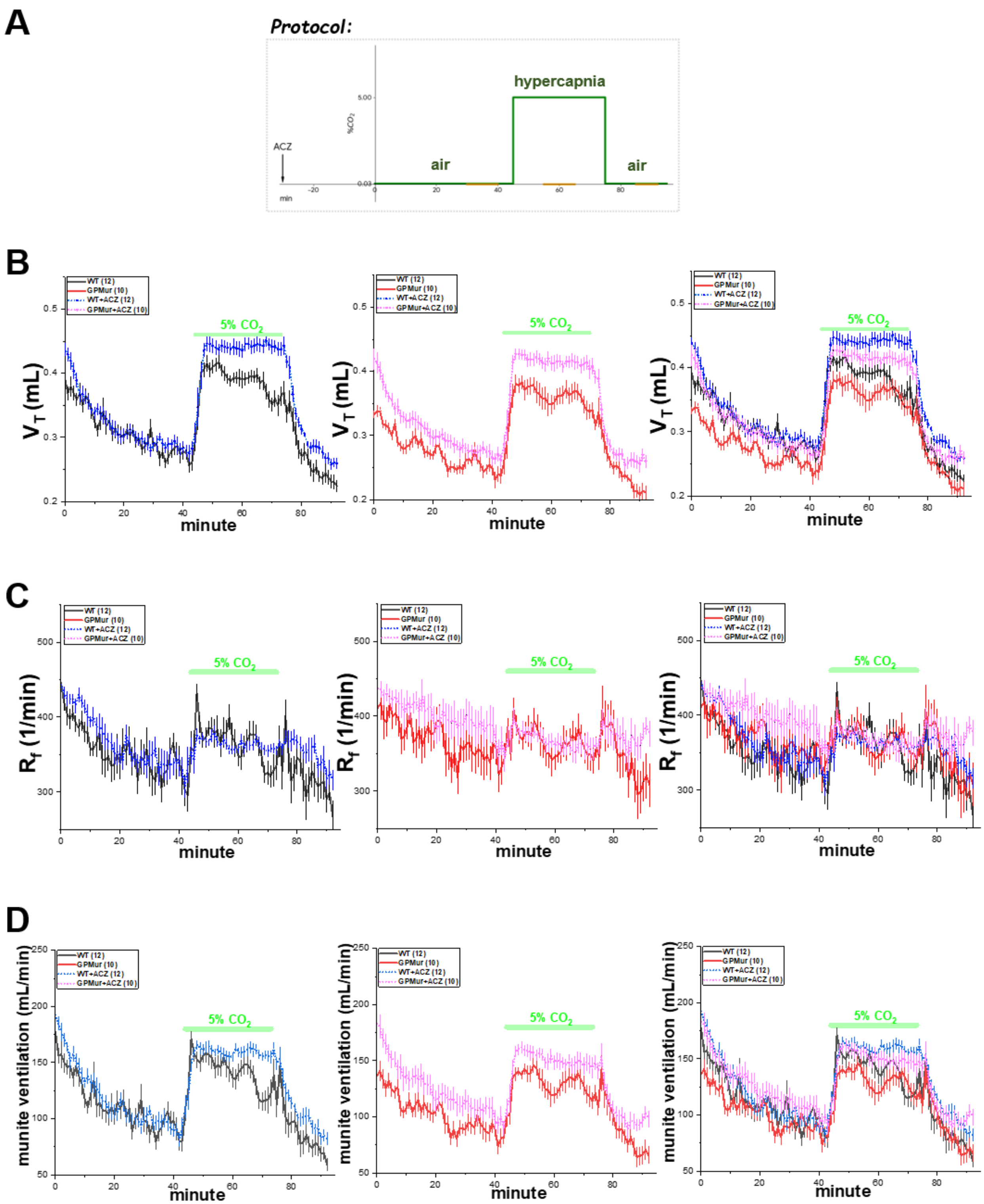

<u>Supplemental Figure 2.</u> Hypercapnia and acetazolamide significantly increased PEF throughout the entire 30-minute gas-in with 5% CO_2 (A). In contrast, PIF was not affected by acetazolamide. GPMur expression mildly lowered PIF.

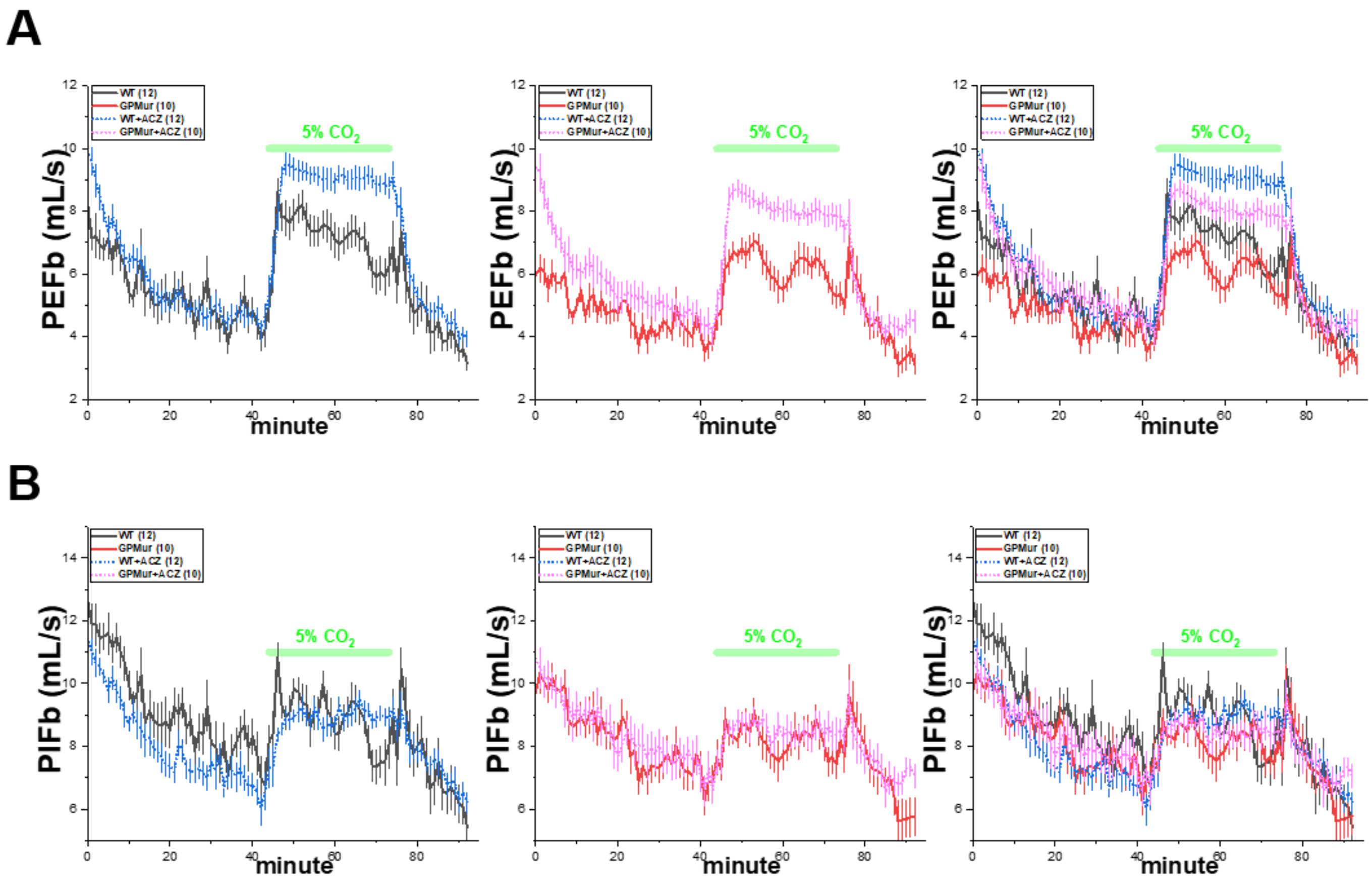

Supplemental Figure 3. Constriction indices Penh and PAU were both increased by hypercapnia and acetazolamide challenges, and reduced with the phenotype of GPMur/increased AE1. Relaxation time (T_r) and the remaining T_e after subtraction of T_r ($T_e - T_r$) are used to calculate pause (PAU), which is the ratio of time spent in the two expiratory phases. Penh, or enhanced PAU, is calculated by multiplying PAU with the ratio of PEP to PIP (PAU * (PEP/PIP)).

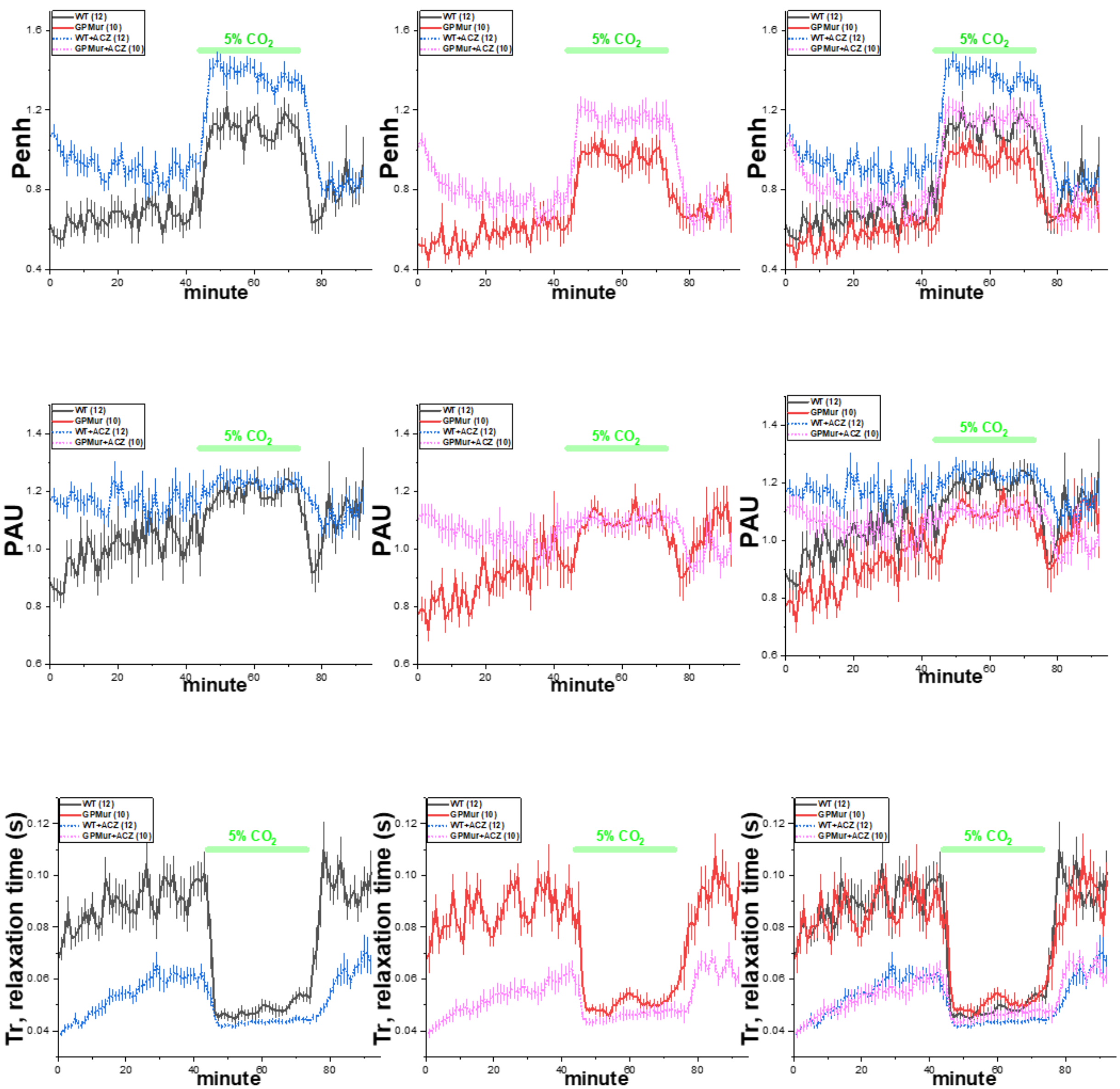

<u>Supplemental Figure 4.</u> Hypercapnia and acetazolamide triggered respiratory acidosis and metabolic acidosis; both decreased expiratory time (T_e) and expiratory duty cycle (T_e/T_{tot})(A-B), and increased inspiratory time (T_i) and inspiratory duty cycle (T_i/T_{tot})(C-D).

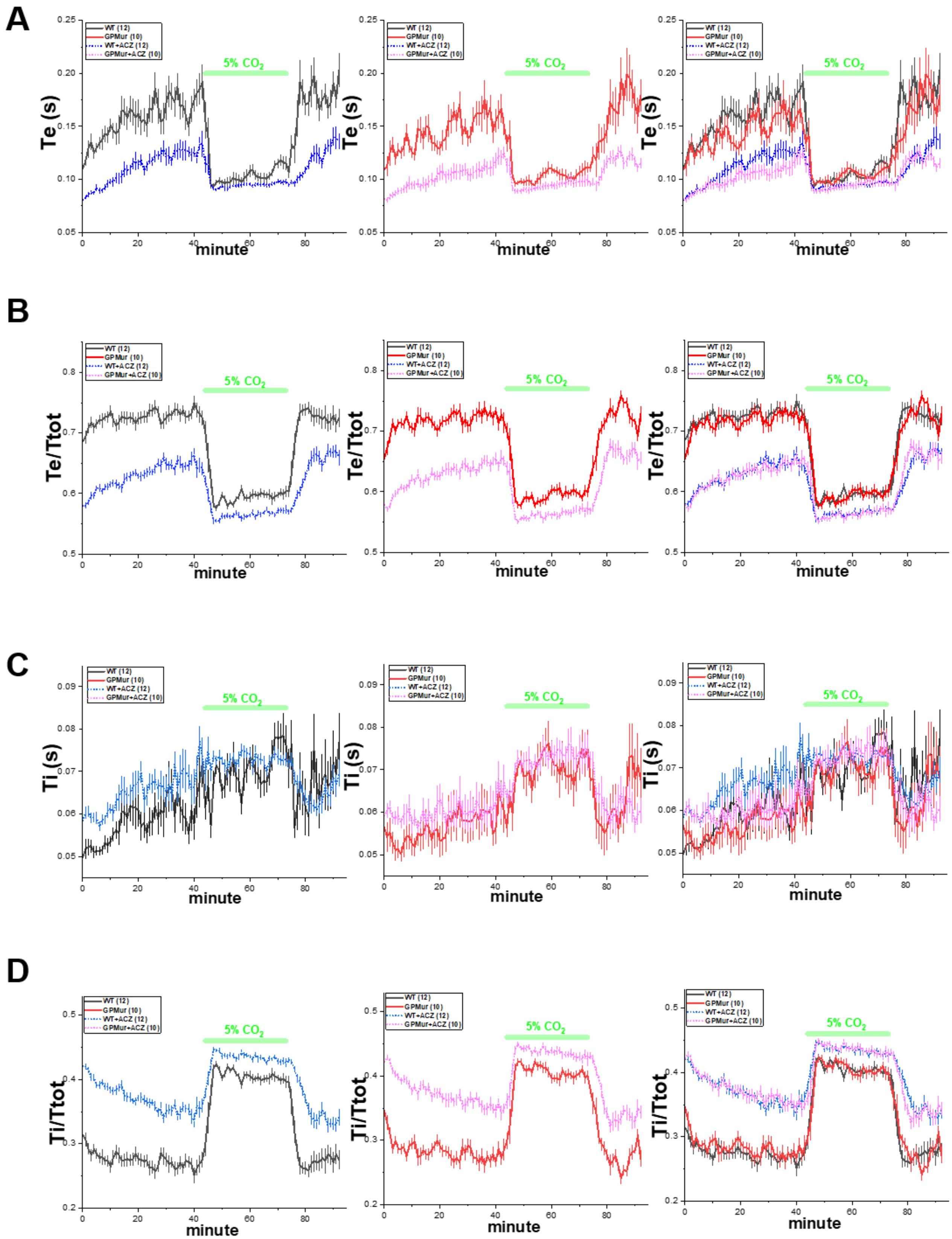

<u>Supplemental Figure 5.</u> Both hypercapnia and acetazolamide challenges increased expiratory drive (VT/T_e); but only hypercapnia, not acetazolamide, promoted inspiratory drive (VT/T_i).

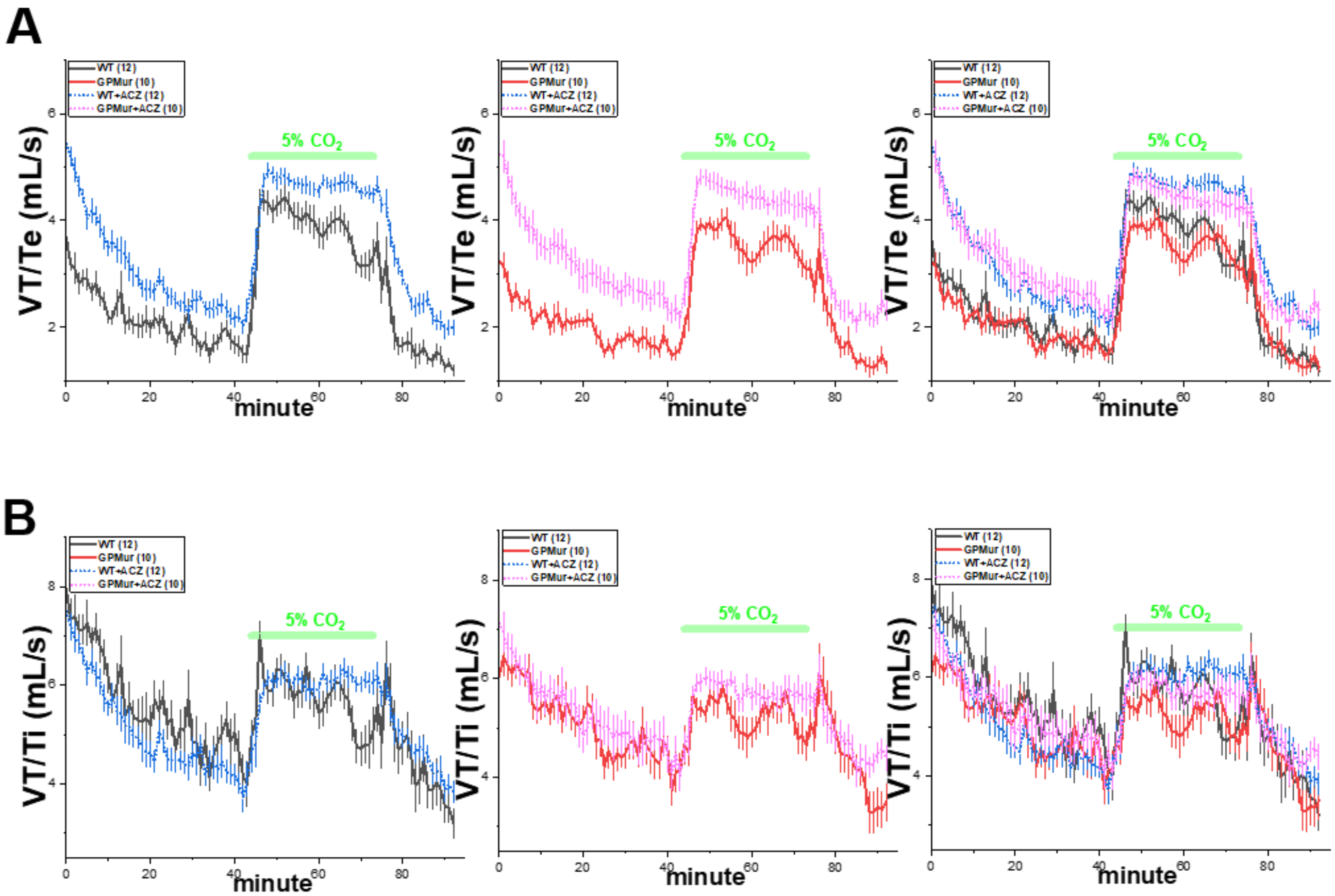

<u>Supplemental Figure 6.</u> Hypercapnia significantly shortened EEP for the entire 30 minutes (A) and transiently prolonged EIP (B). For comparison, acetazolamide substantially shortened EEP and increased EIP with sustaining effects, unlike the transient effects of hypercapnia on EIP.

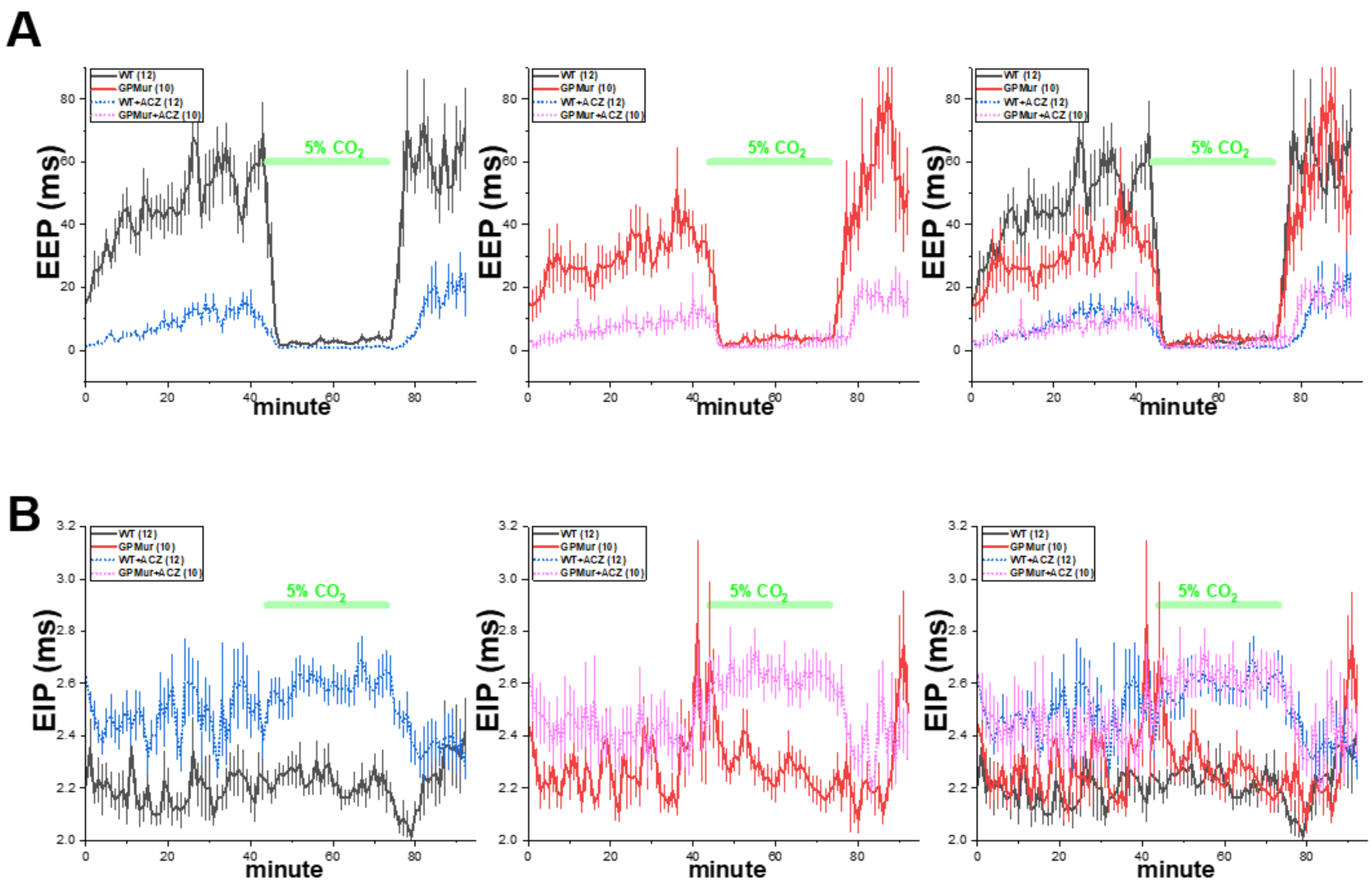

Supplement: Supplementary file 1 [file DataSheet1.pdf]
